# Disease-associated microbiome signature species in the gut

**DOI:** 10.1093/pnasnexus/pgae352

**Published:** 2024-08-21

**Authors:** Junhui Li, Paul W O’Toole

**Affiliations:** APC Microbiome Ireland, University College Cork, Cork T12 K8AF, Ireland; School of Microbiology, University College Cork, Cork T12 K8AF, Ireland; APC Microbiome Ireland, University College Cork, Cork T12 K8AF, Ireland; School of Microbiology, University College Cork, Cork T12 K8AF, Ireland

**Keywords:** signature species, gateway, body site, bacteremia, aerotolerance

## Abstract

There is an accumulation of evidence that the human gut microbiota plays a role in maintaining health, and that an altered gut microbiota (sometimes called *dysbiosis*) associates with risk for many noncommunicable diseases. However, the dynamics of disease-linked bacteria in the gut and other body sites remain unclear. If microbiome alterations prove causative in particular diseases, therapeutic intervention may be possible. Furthermore, microbial signature taxa have been established for the diagnosis of some diseases like colon cancer. We identified 163 disease-enriched and 98 disease-depleted gut microbiome signature taxa at species-level resolution (signature species) from 10 meta-analyses of multiple diseases such as colorectal cancer, ulcerative colitis, Crohn's disease, irritable bowel syndrome, pancreatic cancer, and COVID-19 infection. Eight signature species were enriched and nine were depleted across at least half of the diseases studied. Compared with signature species depleted in diseases, a significantly higher proportion of disease-enriched signature species were identified as extra-intestinal (primarily oral) inhabitants, had been reported in bacteremia cases from the literature, and were aerotolerant anaerobes. These findings highlight the potential involvement of oral microbes, bacteremia isolates, and aerotolerant anaerobes in disease-associated gut microbiome alterations, and they have implications for patient care and disease management.

**Significance statement**

An altered gut microbiome is a feature of many multifactorial diseases. Here we confirm the microbial taxa enriched or depleted in multiple diseases across over 36,800 gut microbiomes. We identify abundance changes in gut microbial taxa that are shared by or specific to, diverse conditions, and we highlight the role of microbial properties that facilitate exchange between body sites implicated in dysbiosis and bacteremia.

## Introduction

Two decades of research have identified many significant associations between altered configurations of the human gut microbiome and diverse noncommunicable diseases (1). Microbial signatures in the gut that are consistently altered across disease cohorts, referred to here as signature taxa, have been observed in specific diseases (2–5). The gut microbiome has been hypothesized to act as an environmental factor in the development of multifactorial diseases and has therefore emerged as a potential therapeutic target. However, it is unclear whether microbiome alterations cause disease or vice versa, or if both are caused by a third factor (6). Understanding the mechanisms that drive microbiome alterations remains a central goal in human microbiome research. Growing evidence supports the notion that the translocation of oral microbes to the gut may contribute to the pathogenesis of chronic diseases (7–11), particularly periodontitis-related oral microbes which may induce dysbiosis in the gut microbiota following their introduction by swallowing (12) or from the blood circulatory system (13). In healthy subjects, microbes may translocate from other body sites into the bloodstream during daily activities such as conducting oral hygiene, following minor medical procedures, or skin injuries (14–16). Bacteremia can progress to a bloodstream infection when the immune response mechanisms are compromised or overwhelmed and might lead to more serious complications (16). Bacteremia is associated with an increased risk of a subsequent diagnosis of colorectal cancer (CRC) (17, 18). Additionally, hospitalized patients with inflammatory bowel disease (IBD) have a higher risk of bacteremia compared with patients without IBD (19).

Understanding the link between the altered gut microbiome, particularly microbial signature taxa, and host effect is crucial for understanding human health and disease. Recent meta-studies have identified reproducible disease-specific microbial signatures across cohorts and populations (2–4), commonly characterized by the depletion of commensals associated with healthy subjects and enrichment of disease-associated pathobionts (20). Two meta-analyses reported disease-specific and overlapping gut microbial signatures at the genus-level across different diseases (5, 21). However, genus-level microbial signatures are not sufficiently robust, because species in the same genus can show contrasting phenotypes (22). High-resolution microbial signatures allow for the exploration of microorganism characteristics (e.g. body site niche, capacity for bloodstream infections, and aerotolerance), which are valuable for understanding disease pathogenesis and for developing microbiome-targeted therapies. A meta-analysis identified common and disease-specific microbiome signature species between CRC and IBD; however, it has focused on developing predictive models for disease diagnosis (23). To advance our understanding of the potential gateways whereby bacteria gain access to the gut, we identified robust microbial signature species from 10 meta-analyses of the gut microbiome associated with different diseases.

## Results and discussion

**Fig. 1.**
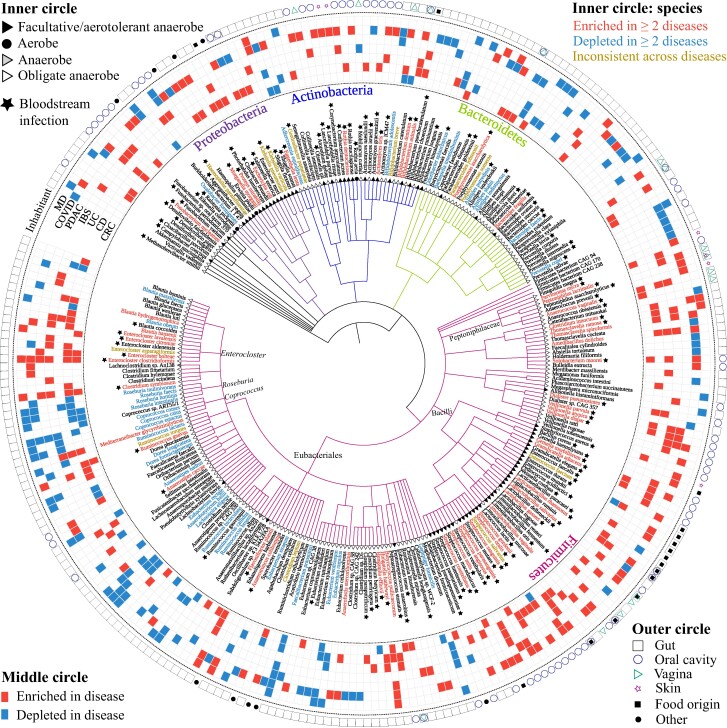
Properties of 273 disease-associated microbial signature taxa based on data extracted from 10 meta-analysis studies. Inner circle: species with names in red text indicate they are enriched in at least two diseases; species names in blue text indicate those depleted in at least two diseases; species names in brown text indicate inconsistent signature taxon across diseases. Solid star in black after taxon name indicates the species was detected in the bloodstream; solid circle at the end of node branch indicates aerobe; solid triangle in black at the end of node branch indicates facultative/aerotolerant anaerobe or; hollow triangle at the end of node branch indicates obligate anaerobe; solid triangle in gray at the end of node branch indicates unclassified anaerobe. Middle circle: Red square indicates signature taxon enriched in disease, and blue square indicates signature taxon depleted in disease. CRC, colorectal cancer; CD, Crohn's disease; UC, ulcerative colitis; IBS, irritable bowel syndrome; PC, pancreatic cancer. Outer circle: shape and color indicate typical body site/niche of signature species, with hollow black rectangle for gut, hollow blue circle for oral cavity, hollow green triangle for vagina, hollow purple star for skin, solid black circle for food origin, and solid black rectangle for other. The presence of multiple shapes for a signature species indicates that the signature species is associated with multiple body sites. Data used in Fig. 1 can be found in Table S2.

We compiled a total of 273 unique gut microbial signature species associated with multiple diseases (MD), including CRC, IBD which includes ulcerative colitis (UC) and Crohn's disease (CD), irritable bowel syndrome (IBS), pancreatic cancer (PC), COVID-19, and MD (which was treated here as one disease) (Table S1). We investigated the characteristics of these signature species, namely whether they had been reported in the literature as detected in bloodstream infections (hereinafter referred to as bacteremia), their aerotolerance, their typical body site/niche, and we investigated the proportional distribution of these characteristics between disease-enriched and disease-depleted signature species. Out of the 273 signature species, 163 are enriched in at least one disease, 98 are depleted in at least one disease and 12 show inconsistent abundance directions across diseases (Fig. 1). Among the 163 signature species enriched in diseases, 105 (64.4%) have been reported in cases of bacteremia, 65 (39.9%) are aerotolerant (i.e. facultative/aerotolerant anaerobe or aerobe), 68 (41.7%) are normal extra-intestinal inhabitants, and 51 are normal inhabitants in the oral cavity (Table 1). Notably, 52 signature species are enriched in MD (i.e. ≥2 diseases), and 84.6% of these 52 species are detected in bacteremia, which is significantly higher than the proportion of bacteremia isolates enriched in one single disease (55.0%, *P* = 2.0e−04, Fisher's exact test, Table 1). Of the signature species that are enriched in MD and bacteremia, 19 are oral inhabitants, 19 are gut inhabitants, and one is a normal vaginal inhabitant, suggesting the origin of bacteremia may be gut or oral inhabitants.

**Table 1.** Proportion of enriched and depleted signature species as a function of number of associated diseases number, body site, and aerotolerance.

Category	Disease-enriched signature species			Disease-depleted signature species		
	Total	1 disease	≥2 disease	Total	1 disease	≥2 diseases
Total	163	111	52	98	64	34
Bacteremia	105 (64.4%)	61 (55.0%)	44 (84.6%)	16 (16.3%)	11 (17.2%)	5 (14.7%)
Aerotolerant	65 (39.9%)	45 (40.5%)	20 (38.5%)	9 (9.2%)	9 (14.1%)	0 (0%)
Inhabitant: oral cavity	51 (31.3%)	32 (28.8%)	19 (36.5%)	2 (2.0%)	2 (3.1%)	0 (0%)
Inhabitant: extra-intestinal	68 (41.7%)	47 (42.3%)	21 (40.4%)	7 (7.1%)	7 (10.9%)	0 (0%)

	Odds ratio	*P*-value
Bacteremia–nonbacteremia vs. enriched–depleted signature species	9.2	1.1e−14
Disease number: *1 disease*	5.8	6.6e−07
Disease number: *≥* *2 diseases*	5.5	7.8e−05
Aerotolerance: *aerotolerant*	23.1	1.0e−04
Aerotolerance: *nonaerotolerant*	4.9	2.3e−06
Inhabitant: *extra-intestinal*	9.3	9.8e−03
Inhabitant: *gut*	5.4	3.0e−06
Aerotolerant–nonaerotolerant vs. enriched–depleted signature species	6.5	3.7e−08
Disease number: *1 disease*	4.1	3.1e−04
Disease number: *≥* *2 diseases*	7.6	4.6e−07
Bloodstream infection: *bacteremia*	8.2	2.3e−03
Bloodstream infection: *nonbacteremia*	1.6	4.1e−01
Inhabitant: *extra-intestinal*	9.4	3.8e−02
Inhabitant: *gut*	2.2	1.4e−01
Oral cavity–gut vs. enriched–depleted signature species	30.2	1.6e−12
Disease number: *1 disease*	18.0	2.4e−07
Disease number: *≥* *2 diseases*	Inf	5.5e−06
Bloodstream infection: *bacteremia*	14.5	1.2e−03
Bloodstream infection: *nonbacteremia*	19.3	3.0e−04
Aerotolerance: *aerotolerant*	Inf	5.9e−04
Aerotolerance: *nonaerotolerant*	13.2	2.0e−05
Extra-intestinal–gut vs. enriched–depleted signature species	11.5	5.5e−12
Disease number: *1 disease*	7.6	4.5e−07
Disease number: *≥* *2 diseases*	Inf	1.1e−06
Bloodstream infection: *bacteremia*	9.6	1.3e−03
Bloodstream infection: *nonbacteremia*	5.5	1.7e−03
Aerotolerance: *aerotolerant*	35.0	7.7e−05
Aerotolerance: *nonaerotolerant*	5.5	1.5−04
Bacteremia–nonbacteremia vs. 1 disease – ≥ 2 diseases: *enriched signature species*	0.22	2.0e−04
Bacteremia–nonbacteremia vs. 1 disease – ≥ 2 diseases: *depleted signature species*	1.2	1
Bacteremia–nonbacteremia vs. aerotolerant–nonaerotolerant: *enriched signature species*	7.2	3.3e−07
Bacteremia–nonbacteremia vs. aerotolerant–nonaerotolerant: *depleted signature species*	1.4	6.5e−01
Bacteremia–nonbacteremia vs. oral cavity–gut: *enriched signature species*	4.4	3.5e−04
Bacteremia–nonbacteremia vs. oral cavity–gut: *depleted signature species*	2.3	3.0e−01
Bacteremia–nonbacteremia vs. extra-intestinal–gut: *enriched signature species*	4.1	1.2e−04
Bacteremia–nonbacteremia vs. extra-intestinal–gut: *depleted signature species*	5.7	2.8e−01

Among these multiple disease-associated signature species, *Fusobacterium nucleatum* and *Gemella morbillorum*, which are oral inhabitants, and *Bacteroides fragilis* (a gut inhabitant) are enriched in CRC, and patients who survived bloodstream infections caused by any of these three signature species had a higher risk of subsequent CRC compared with patients without bloodstream infections (17). Among the 98 disease-depleted signature species, only 16 (16.3%) are reported in bacteremia, 9 (9.2%) are aerotolerant, 7 (7.1%) are extra-intestinal inhabitants, and 2 are inhabitants of the oral cavity (Table 1). 34 signature species are depleted in MD, with 14.7% of these being reported in cases of bacteremia. This proportion is lower than the proportion depleted in a single disease (17.2%, *P* = 1, Fisher's exact test, Table 1), which contrasts with the corresponding comparison for disease-enriched signature taxa. This suggests that gut microbial signature taxa enriched in diseases, particularly MD, tend to be detected in bacteremia that may originate from either the oral cavity or gut.

We next compared the proportions of disease-enriched and disease-depleted signature taxa after stratifying them into subgroups based on bacteremia detection, body site, aerotolerance, or the number of associated diseases. The proportion of disease-enriched signature species detected in bacteremia is significantly higher than that of disease-depleted signature species (OR = 9.2, *P* = 1.1e−14, Fisher's exact test, Table 1), irrespective of their typical body site (extra-intestinal: *P* = 9.8e−03; gut: *P* = 3.0e−06), aerotolerance (aerotolerant: *P* = 1.0e−04; nonaerotolerant: *P* = 2.3e−06), and the number of associated diseases (1 disease: *P* = 6.6e−07; ≥2 diseases: *P* = 7.8e−05).

Secondly, the proportion of disease-enriched signature species that are extra-intestinal inhabitants is much higher than that of disease-depleted signature species (OR = 11.5, *P* = 5.5e−12, Fisher's exact test, Table 1), irrespective of whether the tested phenotype is bacteremia (bacteremia: *P* = 1.3e−03; nonbacteremia: *P* = 1.7e−03), aerotolerance (aerotolerant: *P* = 7.7e−05; nonaerotolerant: *P* = 1.5e−04), or the number of associated diseases (1 disease: *P* = 4.5e−07; ≥2 diseases: *P* = 1.1e−06). Likewise, the proportion of disease-enriched signature species that are normal oral inhabitants is significantly higher than that of depleted signature species (OR = 30.2, *P* = 1.6e−12), irrespective of their being detected in bacteremia, their aerotolerance, or the number of associated diseases (*P* < 0.01).

Thirdly, the proportion of disease-enriched signature species that are aerotolerant is significantly higher than that of depleted signature species (OR = 6.5, *P* = 3.7e−08, Fisher's exact test, Table 1), irrespective of the number of associated diseases (1 disease: *P* = 3.1e−04; ≥2 diseases: *P* = 4.6e−07), suggesting that disrupted gut redox homeostasis may facilitate the overgrowth of aerotolerant/facultative anaerobes and may be involved in pathogenesis. Alternatively, intestinal disease may create a more oxygen-rich environment which selects aerotolerant signature species. It is, however, conceivable that some other phenotypic properties that were not investigated could have affected the results. A significantly higher proportion of aerotolerant taxa was also observed among signature species that are detected in bacteremia (*P* = 2.3e−03) or extra-intestinal inhabitants (*P* = 3.8e−02), but not in signature taxa that are nonbacteremia isolates (*P* = 4.1e−01) or intestinal inhabitants (*P* = 1.4e−01). Patients with numerous diseases including IBD or CRC have been found to have an elevated abundance of facultative anaerobes in their feces (10, 24). There are a number of proposed or established methods by which diet may modulate host ability to regulate the availability of respiratory electron acceptors in the intestine such as oxygen and nitrate, which could contribute to modulating the abundance of aerotolerant anaerobes by controlling the luminal environment (24, 25).

Fourth, among disease-enriched signature species, the proportion of aerotolerant signature species detected in bacteremia is much higher than that of nonaerotolerant signature species (OR = 7.2, *P* = 3.3e−07, Fisher's exact test, Table 1). However, no significant difference associated with aerotolerance was found for disease-depleted signature species (*P* = 6.5e−01). Likewise, the proportion of signature taxa that are normal extra-intestinal inhabitants (or normal oral inhabitants) is significantly higher than that of signature species that are normal intestinal inhabitants among signature species enriched in disease but not depleted in disease (enriched: *P* < 0.001; depleted: *P* > 0.2).

Furthermore, we sought to determine whether there were any phylogenetic patterns in these signature species. Remarkably, among the signature species in the Bacilli class, 35 are enriched in disease, three (*Lactobacillus rogosae*, *Ligilactobacillus ruminis*, and *Weissella confuse*) are depleted in disease, and *Enterococcus faecium* shows inconsistent results between CRC and CD. Although there are numerous well-known probiotic species and strains within the representative *Lactobacillus* genus of the Bacilli class, only one species, *L. rogosae* within the *Lactobacillus* genus is identified as a signature species depleted in disease. On the other hand, two *Lactobacillus* spp. (*L. gasseri* and *L. delbrueckii*) are identified as signature taxa enriched in disease (Fig. 1). In addition, signature species within the Peptoniphilaceae family and the *Enterocloster*, *Fusobacterium*, and *Veillonella* genera are consistently enriched in diseases, while signature species from the *Coprococcus* and *Roseburia* genera are consistently depleted in diseases (Fig. 1). Intriguingly, multiple species in the genera *Anaerostipes*, *Bacteroides*, *Bifidobacterium*, *Blautia*, and *Ruminococcus* have been identified as signature species for MD. However, some species within the same genus have been observed to exhibit contrasting associations (Fig. 1). For instance, e.g. *Anaerostipes hadrus* (depleted in MD) vs. *Anaerostipes caccae* (enriched in MD); *Bacteroides faecis* (depleted) vs. *B. fragilis* (enriched); *Bifidobacterium adolescentis* (depleted) vs. *Bifidobacterium animalis* and *Bifidobacterium dentium* (enriched); *Blautia massiliensis* and *Blautia obeum* (depleted) vs. *Blautia hansenii* and *Blautia hydrogenotrophica* (enriched); *Ruminococcus lactaris*, *Ruminococcus bicirculans*, *Ruminococcus bromii*, and *Ruminococcus callidus* (depleted) vs. *Ruminococcus gnavus* (enriched). This suggests the necessity of high-resolution microbial signatures in the future development of gut microbiome diagnostics or therapeutics.

Finally, we examined the gut microbial signature taxa linked to at least half of the diseases and discovered that eight are enriched while nine are depleted in at least half of the diseases. These disease-enriched signature species include *Enterocloster clostridioformis* (6 diseases), *Clostridium symbiosum* (5), *Flavonifractor plautii* (5), *Hungatella hathewayi* (5), *Amedibacillus dolichus* (4), *F. nucleatum* (4), *Streptococcus gordonii* (4), and *Streptococcus mitis* (4). Seven of these eight disease-enriched signature taxa have been detected in bacteremia. The disease-depleted signature species include *Faecalibacterium prausnitzii* (6), *Anaerobutyricum hallii* (5), *Coprococcus comes* (5), *Eubacterium rectale* (4), *Eubacterium ventriosum* (4), *Gemmiger formicilis* (4), *Lachnospira eligens* (4), *Roseburia faecis* (4), and *Roseburia hominis* (4). All nine signature species depleted in at least half of the diseases are nonbacteremia, nonaerotolerant, and normal intestinal inhabitants. These shared microbial signature species include many of the “usual suspects” identified across multiple noncommunicable diseases with microbiome alterations (5, 26, 27), characterized by a depletion of short-chain fatty acid producers and an enrichment of opportunistic pathogens in diseases (Table S1), while the disease-enriched *A. dolichus* (formerly *Eubacterium dolichum*) has been found to be the only species significantly associated with a higher visceral fat mass diet score (28). These gut microbial signature species, which are associated with MD, may serve as more generalized health indicators or therapeutic targets.

Given that four meta-studies associated with CRC (2, 29–31) were included in this work, we further investigated the reproducibility of the microbial signature species identified in the four meta-studies. Of the 55 identified microbial signature species associated with CRC, 36 (65.5%) were identified in a single meta-study, 12 (21.8%) in two meta-studies, 3 (5.5%) in three meta-studies, and only 4 (7.3%) were identified across all four meta-studies (Table S2). These results indicate that there is a low level of signature robustness between the CRC meta-studies. Despite the heterogeneity of the metagenomic sequencing cohorts with regard to laboratory methodology, DNA sequencing, and geographic region, this is less likely to be the primary factor contributing to the low comparability between meta-studies, given that the majority of the cohorts included in each meta-study have been included in other CRC meta-studies as well (https://github.com/junhuili/gut_biomarkers/blob/main/supplementary_information.xlsx). The bioinformatics pipelines and methodologies (e.g. covariate adjustment, differential abundance analysis, mixed-effect model, random effects meta-analysis models, machine learning model) employed for the identification of signature species across cohorts may be a significant contributing factor to the discrepancy between the CRC meta-studies. Our study highlights the critical necessity for a meta-meta-analysis wherein all data would be processed in a highly standardized way.

This study was limited by compiling microbial signature species from meta-studies that included heterogeneous cohorts with regard to the lab methodology and DNA sequencing, particularly bioinformatics pipelines and methodologies employed for identifying microbial signature species. One limitation of relying on literature-reported bloodstream infections for microbial signature taxa is that the reported isolation does not necessarily infer causative bloodstream infections in the patients. Rather, it simply indicates the signature taxon was detected as present in the blood in certain disease cases. Another limitation is that certain signature species associated with multiple habitats or with unknown aerotolerance were excluded in the respective statistical analyses. A further limitation in the potential usage of our study findings for the prevention and treatment of microbiome-related diseases is the lack of longitudinal characterization within each patient, which hinders our ability to determine the variability in presentation of a signature species. Furthermore, a significant challenge is the lack of quantitative microbiome profiling, which limits the ability to elucidate the interplay between microbiome features and host health. Further meta-analysis is warranted when such data become available to evaluate the variation of signature species.

In summary, our findings reveal that a greater proportion of gut microbial signature taxa enriched in diseases are typically extra-intestinal (primarily oral) inhabitants, are detected in bloodstream infections as reported in the literature, and are aerotolerant, compared with signature taxa depleted in diseases. The emerging picture further supports the notion [reviewed in O’Toole et al. (27)] that establishment of oral inhabitants in the gut may potentially contribute to microbiome alterations and associated diseases, and highlights the importance of evaluating the gut as a source of bloodstream infections in clinical practice. A holistic understanding of within-subject microbiome dynamics may lead to opportunities for prospective risk reduction of noncommunicable microbiome-related diseases.

## Materials and methods

### Meta-studies included for compiling gut signature species associated with disease

To identify microbial signature taxa associated with diseases, we compiled data from 10 meta-studies aiming to identify reproducible disease-associated gut microbial signatures at the species-level across multiple case-control cohorts in April 2023. Meta-studies that identified signature taxa bellow the species-level for the listed diseases were excluded. These diseases include CRC (2, 29–31), IBD viz. UC and CD (32), IBS (3), PC (4), COVID-19 infection (33, 34), and MD (which was considered as one disease) (35) (Table S1). The individual cohorts included in each meta-analysis can be found at https://github.com/junhuili/gut_biomarkers/blob/main/supplementary_information.xlsx.

### Criteria for defining the body site, bacteremia, and aerotolerance of microbial signature species

The modified criteria for defining oral bacteria (8) were used to determine the body site of microbial signature species. We calculated the frequency and the average relative abundance of signature species across four human body sites (gut, oral cavity, vagina, and skin) in the NIH Human Microbiome Project (HMP1) using HMSMCP2—metagenomic taxon abundances (https://www.hmpdacc.org/hmsmcp2/), and the body site that had the highest frequency and average relative abundance of the signature species was designated as the body site for the signature species. For signature species meeting any of the following conditions: a highest frequency of less than 5% across four body sites, either the highest frequency or the highest average relative abundance (but not both), a gut/oral cavity ratio between 0.1 and 10, and not detected in the HMSMCP2, further considerations were taken into account, including information concerning the signature species in the expanded Human Oral Microbiome Database v3.1 (36), the frequency and the average relative abundance of signature species in the gut shotgun metagenomes of healthy individuals in ExperimentHub (*n* = 12,788) (37) and American Gut Project (*n* = 229) (38), and/or the isolation source of signature species from NCBI genomes (https://github.com/junhuili/gut_biomarkers/blob/main/supplementary_information.xlsx) and literature references. See Table S2 for detailed information of the normal body site for each signature taxon.

To determine if the signature species are detected in cases of bacteremia, we searched several databases including MEDLINE, PubMed, Scopus, Web of Science, and Google Scholar using the terms “signature taxon name” AND “bacteremia” and “signature taxon name” AND “bloodstream infection” in September 2023. The search included both current and former names of the signature species, with or without a genus abbreviation. If a patient has positive blood cultures associated with a certain disease, infection, or symptom, the signature species was considered as bloodstream infection (or bacteremia). Additionally, we conducted a thorough search of these databases to investigate the aerotolerance of signature species: aerotolerant (facultative/aerotolerant anaerobe, aerobe) or nonaerotolerant (obligate anaerobe). The aerotolerance of five signature taxa, *Anaeromassilibacillus* sp. An250, *Lachnoclostridium* sp. An138, *Firmicutes bacterium* CAG 170, *F. bacterium* CAG 238, and *F. bacterium* CAG 94, is unknown.

### Statistical analysis

Fisher's exact test was applied to evaluate the significant difference in feature proportions between different conditions. Signature species associated with multiple body sites or without known aerotolerance levels were not included in the statistical analyses. The phylogenetic tree, which includes all signature species, was obtained from NCBI common tree and visualized using iTOL v6 (39).

## Supplementary Material

pgae352_Supplementary_Data

## Data Availability

All data relevant to the study are available in the main text or in the Supplementary material. The sources supporting the characteristics of signature taxa have been deposited in https://github.com/junhuili/gut_biomarkers/.
